# Cystatin C to Left Ventricular Ejection Fraction Ratio as a Novel Predictor of Adverse Outcomes in Patients with Coronary Artery Disease: A Prospective Cohort Study

**DOI:** 10.31083/j.rcm2409260

**Published:** 2023-09-18

**Authors:** Yi Ning, Kai-Yang Wang, Xuan Min, Xian-Geng Hou, Ting-Ting Wu, Yi-Tong Ma, Xiang Xie

**Affiliations:** ^1^Department of Cardiology, First Affiliated Hospital of Xinjiang Medical University, 830011 Urumqi, Xinjiang, China

**Keywords:** cystatin C, left ventricular ejection fraction, outcomes, coronary artery disease

## Abstract

**Background:**

While both cystatin C and left ventricular ejection fraction (LVEF) revealed established prognostic efficacy in coronary artery disease (CAD), the relationship between cystatin C/left ventricular ejection fraction ratio (CLR) and adverse clinical outcomes among patients with CAD following percutaneous coronary intervention (PCI) remains obscure, to date. Therefore, we sought to assess the predictive efficacy of CLR among CAD patients who underwent PCI in current study.

**Methods:**

A total of 14,733 participants, including 8622 patients with acute coronary syndrome (ACS) and 6111 patients with stable coronary artery disease (SCAD), were enrolled from a prospective cohort of 15,250 CAD patients who underwent PCI and were admitted to the First Affiliated Hospital of Xinjiang Medical University from 2016 to 2021. The primary outcome of this study was mortality, including all-cause mortality (ACM) and cardiac mortality (CM). The secondary outcomes were major adverse cardiovascular events (MACEs), major adverse cardiac and cerebrovascular events (MACCEs) and nonfatal myocardial infarction (NFMI). For CLR, the optimal cut-off value was determined by utilizing receiver operating characteristic curve analysis (ROC). Subsequently, patients were assigned into two groups: a high-CLR group (CLR ≥0.019, n = 3877) and a low-CLR group (CLR <0.019, n = 10,856), based on optimal cut-off value of 0.019. Lastly, the incidence of outcomes between the two groups was compared.

**Results:**

The high-CLR group had a higher incidence of ACM (8.8% vs. 0.9%), CM (6.7% vs. 0.6%), MACEs (12.7% vs. 5.9%), MACCEs (13.3% vs. 6.7%), and NFMIs (3.3% vs. 0.9%). After adjusting for confounders, multivariate Cox regression analyses revealed that patients with high-CLR had an 8.163-fold increased risk of ACM (HR = 10.643, 95% CI: 5.525~20.501, *p* < 0.001), a 10.643-fold increased risk of CM (HR = 10.643, 95% CI: 5.525~20.501, *p* < 0.001), a 2.352-fold increased risk of MACE (HR = 2.352, 95% CI: 1.754~3.154, *p* < 0.001), a 2.137-fold increased risk of MACCEs (HR = 2.137, 95% CI: 1.611~2.834, *p* < 0.001), and a 1.580-fold increased risk of NFMI (HR = 1.580, 95% CI: 1.273~1.960, *p* < 0.001) compared to patients with low-CLR.

**Conclusions:**

The current study indicated that a high CLR is a novel and powerful predictor of adverse long-term outcomes in CAD patients who underwent PCI, and that, it is a better predictor for patients wtih SCAD and ACS.

**Clinical Trial Registration:**

NCT05174143, http://Clinicaltrials.gov.

## 1. Introduction

Coronary artery disease (CAD) is the leading cause of death and morbidity related to cardiovascular diseases worldwide [1]. In China, the incidence of CAD is increasing annually [2]. Although several predictors of CAD-related death have been reported [3, 4, 5, 6], more powerful predictors need to be developed.

Cystatin C (Cys-C) is produced by all nucleated cells regardless of age, sex, muscle mass or diet, making it one of the best indicators of renal function [7, 8]. Nevertheless, the characteristics of Cys-C as an inhibitor of cysteine proteases make it relevant to atherosclerosis and cardiovascular disease [9, 10]. It was reported recently that Cys-C increased the incidences and worse outcomes of acute coronary syndrome (ACS) [11], cardiac insufficiency [12] and acute kidney injury (AKI) [13]. Several studies have also indicated that Cyc-C contributes to cardiovascular risk and inflammation [14, 15]. Among patients with CAD, Cyc-C serves as an important biomarker of long-term mortality from all causes and cardiovascular disease [16].

It is well known that left ventricular ejection fraction (LVEF) is widely recognized as a measure of heart function. Patients with cardiovascular disease and heart failure with lower LVEF values have a higher mortality rate [17]. Patients with reduced LVEF and heart failure were significantly more likely to die and suffer myocardial infarction at 3 years than those with heart failure and mild to moderate LVEF [18].

In recent studies, either Cys-C or LVEF has been independently linked to cardiovascular disease (CVD) and mortality [9, 10]. In spite of this, there is no consensus on the usefulness of Cys-C/LVEF ratio (CLR) for predicting adverse outcomes in CAD patients. Since Cys-C and the LVEF enhances coronary artery disease progression and can evaluate coronary artery lesions’ severity [14, 15, 17], there is a reasonable possibility of predicting the performance of CLR in CAD patients. Hence, a prospective cohort study, which is consisted of 15,250 CAD patients who underwent PCI and long-term follow-ups were conducted, was designed to explore the relevance between CLR and adverse outcomes.

## 2. Methods

### 2.1 Study Design and Population

Patients enrolled in current study were all receiving Personalized Antiplatelet Therapy based on the Genotype of CYP2C19 (PRACTICE) for CAD, a study conducted in the First Affiliated Hospital of Xinjiang Medical University based on patients with unabridged case records and follow-up registries from 2016 to 2021. A clinical trial registration number has been assigned to the design (identifier: NCT05174143) at http://Clinicaltrials.gov. Our study population included only PRACTICE participants with inclusion criteria: (1) detailed clinical histories; (2) explicit diagnosis of CAD, including non-ST-segment elevation acute coronary syndrome (ACS), ST-segment elevation myocardial infarction (STEMI) and stable angina, with stenosis ≥50% on coronary angiography or computed tomography angiography (CTA) and at least one stent implantation was performed. Patients with valvular heart disease, rheumatic heart disease, congenital heart disease, pulmonary heart disease, haematological diseases, malignant tumours, and organ malfunction such as the liver or kidneys were excluded.

**Fig. 1.**
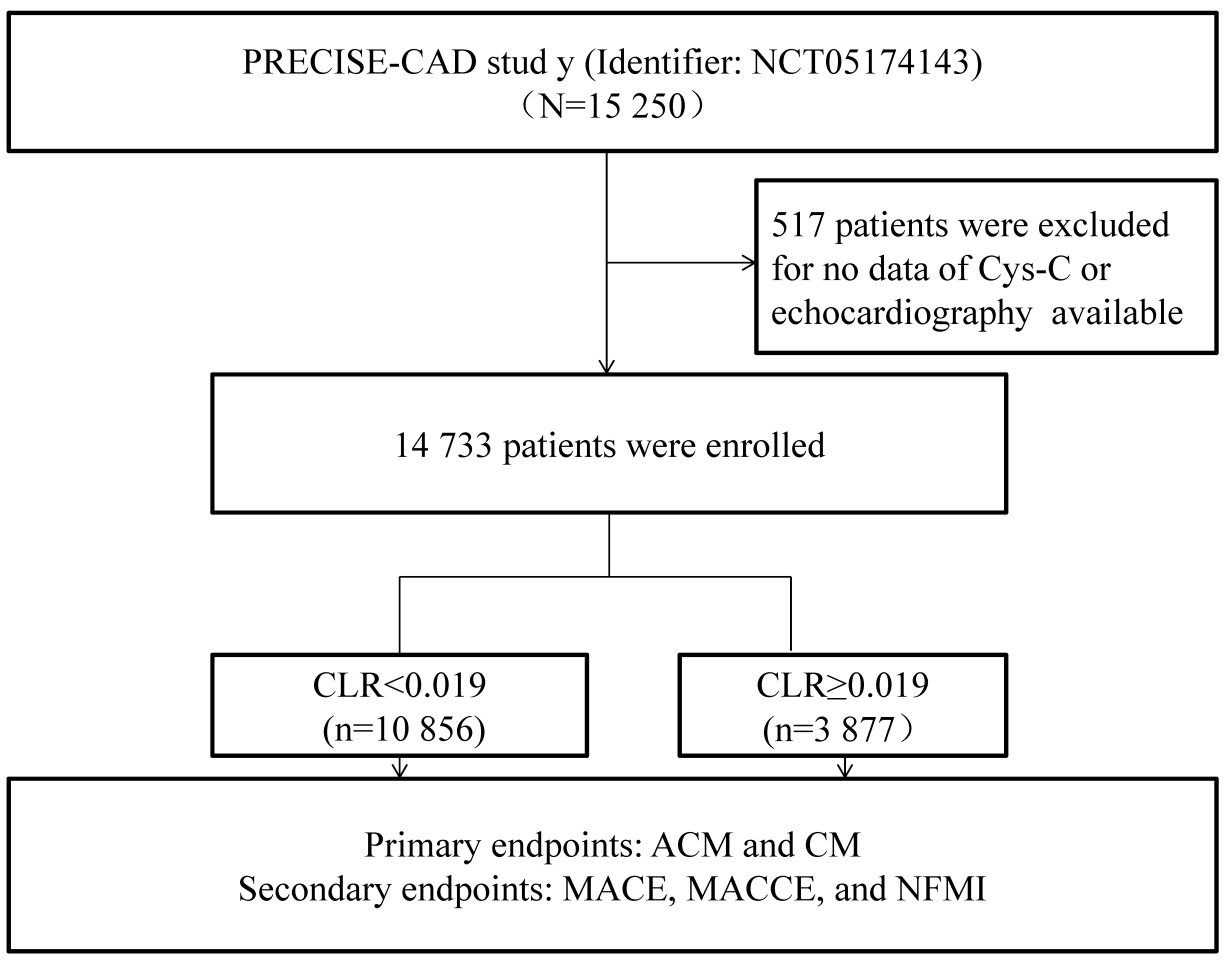
**Overview of the inclusion process**. Abbreviations: CAD, coronary artery disease; ACM, all-cause mortality; CM, cardiac mortality; MACE, major adverse cardiovascular event; MACCE, major adverse cardiovascular and cerebrovascular event; NFMI, nonfatal myocardial infarction; Cys-C, cystatin C; CLR, cystatin C/left ventricular ejection fraction ratio.

Initially, 15,250 CAD patients were evaluated to determine the relevance between CLR and PCI outcomes, in which 517 were excluded on account of the absence of echocardiography or Cys-C data. Ultimately, 14,733 were enrolled, including 8622 patients with ACS and 6111 patients with stable coronary artery disease (SCAD). The detailed inclusion and exclusion criteria were illustrated in Fig. 1 by a flowchart. Ethics Committee approval was granted for the study protocol from First Affiliated Hospital of Xinjiang Medical University, and informed consent was waived.

### 2.2 Endpoints and Follow-Up

Patients who underwent PCI at our center were regularly followed up after discharge for 1, 3, and 6 months, and then for 1, 3, and 5 years, and the median follow-up time was 24 (1–60) months in this study. Following up with patients was done by either outpatient interviews or telephone calls as required. All events were reviewed and checked by a group of experienced clinical physicians comprehensively during the follow-up period. To ensure that we obtained high-quality data, we trained the investigators before the start of the study. In order to ensure consistency, all questionnaires were completed blindly, and telephone follow-ups were conducted as per a uniform set of rules. An evaluation of medication adherence and adverse events was conducted at all clinical follow-ups. A primary outcome of this study was mortality including all-cause mortality (ACM) and cardiac mortality (CM), while strokes, nonfatal myocardial infarction, and bleeding events were considered as secondary outcomes. The major adverse cardiac events (MACEs) were defined as cardiac death and nonfatal myocardial infarction (NFMI), and major adverse cardiac cerebrovascular events (MACCEs) were defined as a combination of cardiac death, NFMI, and stroke.

### 2.3 Data Collection

Data on PCI procedures, demographics, clinical characteristics, cardiovascular risk factors, echocardiography, laboratory testing, and short-/long-term outcomes were all collected and recorded. Factors associated with cardiovascular disease include smoking, alcohol consumption, diabetes, and hypertension. The diagnostic criteria of diabetes mellitus consisted of a history of diabetes and regular intake of antidiabetic drugs, or a fasting plasma glucose of ≥7.7 mmol/L, or a two-hour post-load glucose of ≥11.1 mmol/L [5], while hypertension was defined as a blood pressure ≥140/90 mmHg which was measured repeatedly (at least three times at different resting positions) and treated regularly with antihypertensive drugs [6]. Body mass index (BMI) was calculated by dividing the weight in kilograms by the height in metres squared. The patients were categorized as current smokers, former smokers, or never smokers based on their smoking status. Those who smoked regularly over the past six months were regarded as current smokers, and those consumed alcohol usually over the past six months were regarded as alcohol users. We also collected medication information and medical history. In all the PRACTICE patients, the LVEF and left ventricular end diastolic diameter (LVEDD) were measured on admission according to the American Society Echocardiography guidelines. Trained hospital personnel used a Philips ultrasonic instrument to perform echocardiography examinations for all the patients in accordance with a standard imaging protocol. Digital loops and images were recorded in the left recumbent position for all subjects. Parameters including the diameters of the atrium and ventricle, pulmonary artery pressure, and LVEF values were recorded. To calculate the LVEF, a simple formula was used: EF = ((EDV–ESV)/EDV) × 100% according to Biplane Simpson’s rule, which was evaluated as a continuous and dichotomous variable. An LVEF <50% was defined as LV systolic dysfunction [18, 19]. Immunoturbidimetry was used to measure serum Cys-C levels as previously described. The glomerular filtration rate (eGFR) was taken to assess renal function, with impaired renal function being defined as an eGFR of less than 60 mL/min/1.73 $m^{2}$, and creatinine was calibrated using the Jaffe dynamic method. To estimate eGFR, the Chinese version of Modification of Diet in Renal Disease equation (C-MDRD) was utilized [10, 12, 20]. Laboratory test data, including routine blood test parameters, fasting serum concentration of uric acid, liver function, renal function, myocardial enzyme profile, lipid profile, and glucose, were tested via classic methods in the Central Laboratory of the First Affiliated Hospital of Xinjiang Medical University, as described previously [21]. Only the first measurement was included.

### 2.4 Statistical Analyses

SPSS 22.0.1 for Windows (SPSS Inc., Armonk, NY, USA) was utilized for data analyses. Continuous values are presented as the mean ± standard deviation (SD). A normality test was carried out before the data analyses. Numerical variables with normally distributed distributions were analyzed by the student *t* test, the analyses of non-normally distributed variables were done by the Mann-Whitney U test, and a chi-square test (χ^2^) was executed to compare categorical variables. For CLR, the optimal cut-off value was determined by utilizing receiver operating characteristic curve analysis (ROC). To calculate cumulative survival curves, Kaplan-Meier analysis was performed followed by the log-rank test. The predictability of the CLR was evaluated using a Cox proportional risk regression model, with hazard ratios and 95% confidence intervals. *p* < 0.05 was considered statistically significant.

## 3. Results

### 3.1 The Optimal Cut-Off Value of the CLR

**Fig. 2.**
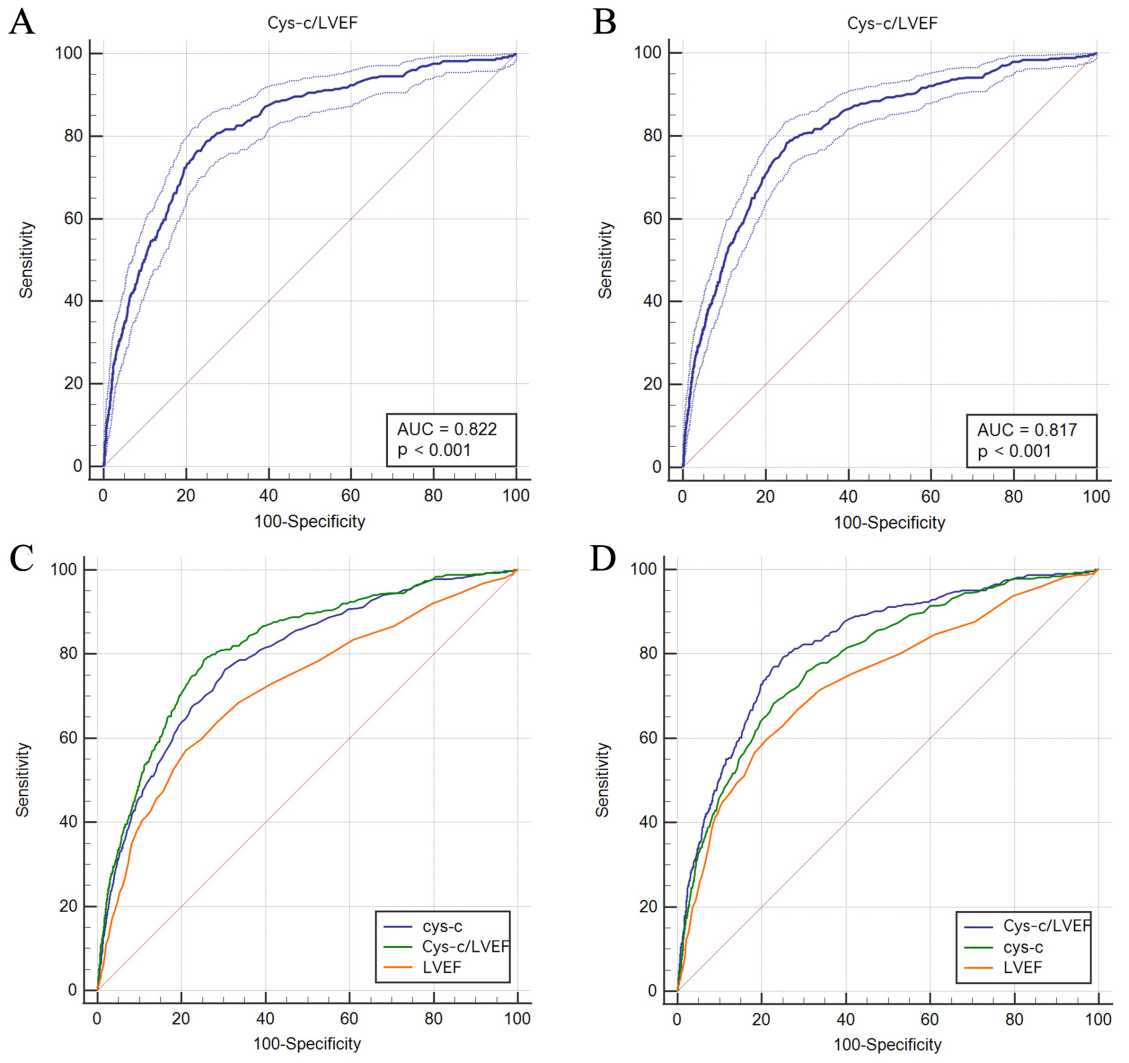
**ROC analysis of the predictability of CLR for ACM (A) and CM (B) and comparison among Cys-C, CLR, and LVEF in terms of ACM (C) and CM (D)**. ACM, all-cause mortality; CM, cardiac mortality; LVEF, left ventricular ejection fraction; Cys-C, Cystatin C; CLR, Cys-C/LVEF ratio; ROC, receiver operating characteristic curve analysis.

To set multiple critical values for the continuous variable CLR, a series of sensitivity and specificity values were calculated via ROC curve analysis. The ordinate of the curve was sensitivity, while the abscissa of the curve was 1-specificity. In this study, the optimal cut-off point for the CLR was 0.019 (AUC = 0.817, 95% CI: 0.811–0.823, *p* < 0.001) in ACM, with high sensitivity (78.33% in ACM, 78.72% in CM) and specificity (74.78% in ACM, 75.10% in CM), Fig. 2A; (AUC = 0.822, 95% CI: 0.816–0.828, *p* < 0.001 in CM, Fig. 2B), which located at the upper left of the coordinate plot. Subsequently, patients were assigned into low-CLR group (CLR <0.019, n = 10,856) and high-CLR group (CLR ≥0.019, n = 3877) based on the optimal cut-off value of CLR (0.019). Furthermore, the areas under the curve (AUCs) among Cys-C, LVEF, and CLR were compared. The AUCs of the CLR were significantly higher than those of Cys-C or LVEF alone for both ACM (0.819 vs. 0.790 vs. 0.719, *p* < 0.001, Fig. 2C) and CM (0.826 vs. 0.790 vs. 0.739, *p* < 0.001, Fig. 2D).

### 3.2 Baseline Data

Overall, no significant differences were observed between the two groups in terms of β-blocker therapy, clopidogrel, blood urea nitrogen (BUN) or uric acid (all *p* > 0.05). Nevertheless, several significant differences were observed between the two groups, including sex, smoking, drinking, hypertension, diabetes, age, BMI, serum creatinine (SCr), eGFR, LVEDD, total cholesterol (TC), high-density lipoprotein (HDL-C), low-density lipoprotein (LDL-C), LVEF, Cys-C and therapy with angiotensin receptor blocker (ARB) or angiotensin-converting enzyme inhibitor (ACEI), calcium channel blocker (CCB), aspirin and statins (all *p* < 0.05, as shown in Table 1). In addition, smoking, drinking, age, BUN, SCr, eGFR, TC, HDL-C, LDL-C, LVEF, LVEDD, Cys-C, ARB or ACEI and statins were found to be different between the two groups in patient with SCAD, while sex, smoking, drinking, age, eGFR, TC, HDL-C, LDL-C, LVEF, LVEDD, Cys-C, ARB or ACEI, CCB, aspirin and statins were significantly different between the two groups in patients with ACS (all *p* < 0.05, as shown in Table 1).

**Table 1.** **Comparison of baseline characteristics between the two groups**.

Characteristic	Low-CLR	High-CLR	χ^2^*/t*	*p*-values
Total (n = 14,733)	n = 10,856	n = 3877		
Male sex, n (%)	7953 (73.3)	2934 (75.7)	8.659	0.003
Smoking, n (%)	4395 (40.5)	1451 (37.4)	11.166	0.001
Drinking, n (%)	2708 (24.9)	784 (20.2)	35.237	<0.001
Hypertension, n (%)	7310 (67.4)	2780 (72.2)	31.173	<0.001
Diabetes, n (%)	4600 (42.4)	2369 (61.1)	402.092	<0.001
Age (years)	58.44 ± 10.92	64.93 ± 11.88	44.922	<0.001
BMI (kg/$m^{2}$)	26.08 ± 3.85	25.67 ± 4.09	12.239	0.006
BUN (mmol/L)	8.92 ± 31.28	9.94 ± 23.34	2.464	0.064
Uric acid (mmol/L)	435.48 ± 611.94	436.09 ± 387.21	51.283	0.958
SCr (µmol/L)	83.55 ± 375.96	97.64 ± 69.32	0.016	<0.001
eGFR (mL/min/1.73 $m^{2}$)	102.10 ± 31.39	82.69 ± 44.57	582.437	<0.001
TC (mmol/L)	3.93 ± 1.10	3.74 ± 1.07	3.236	<0.001
HDL-C (mmol/L)	1.08 ± 0.31	1.01 ± 0.30	2.917	<0.001
LDL-C (mmol/L)	2.50 ± 0.90	2.39 ± 0.86	12.544	<0.001
LVEF (%)	62.52 ± 5.04	53.53 ± 10.83	5020.597	<0.001
LVEDD (mm)	48.92 ± 4.12	53.34 ± 7.55	1978.634	<0.001
Cys-C (mg/L)	0.84 ± 0.18	1.58 ± 1.16	1378.582	<0.001
ARB or ACEI, n (%)	4509 (41.5)	1849 (47.7)	44.144	<0.001
β-Blockers, n (%)	6046 (57.9)	2114 (58.1)	0.054	0.817
CCB, n (%)	2290 (21.9)	735 (20.2)	4.727	0.030
Aspirin, n (%)	10,387 (95.7)	3631 (93.7)	25.368	<0.001
Statins, n (%)	10,180 (93.8)	3510 (90.5)	45.564	<0.001
Clopidogrel, n (%)	5569 (51.3)	1999 (51.6)	0.078	0.780
SCAD (n = 6111)	n = 4851	n = 1260		
Male sex, n (%)	3533 (72.8)	928 (73.7)	0.342	0.559
Smoking, n (%)	1964 (40.5)	455 (36.1)	8.007	0.005
Drinking, n (%)	1231 (25.4)	254 (20.2)	14.801	<0.001
Age (years)	59.61 ± 10.62	66.67 ± 11.49	11.361	<0.01
BMI (kg/$m^{2}$)	26.28 ± 3.87	25.88 ± 4.07	1.506	0.066
BUN (mmol/L)	9.18 ± 31.61	11.55 ± 31.16	3.226	0.018
Uric acid (mmol/L)	445.70 ± 650.25	426.00 ± 369.63	32.310	0.302
SCr (µmol/L)	77.89 ± 269.99	99.43 ± 70.26	1.360	0.010
eGFR (mL/min/1.73 $m^{2}$)	102.49 ± 30.97	82.19 ± 47.47	244.703	<0.01
TC (mmol/L)	3.92 ± 1.09	3.74 ± 1.08	2.260	<0.01
HDL-C (mmol/L)	1.09 ± 0.30	1.04 ± 0.30	1.774	<0.01
LDL-C (mmol/L)	2.49 ± 0.90	2.38 ± 0.87	5.057	<0.01
LVEF (%)	62.88 ± 4.83	55.24 ± 10.73	1703.314	<0.01
LVEDD (mm)	48.78 ± 4.01	52.41 ± 7.30	619.446	<0.01
Cys-C (mg/L)	0.83 ± 0.18	1.60 ± 1.50	377.134	<0.01
ARB or ACEI, n (%)	2059 (42.4)	606 (48.1)	12.985	<0.001
β-Blockers, n (%)	2771 (59.0)	702 (58.8)	0.014	0.906
CCB, n (%)	1187 (25.3)	322 (26.9)	1.383	0.240
Aspirin, n (%)	4716 (97.2)	1212 (96.2)	3.629	0.057
Statins, n (%)	4575 (94.3)	1164 (92.4)	6.514	0.011
Clopidogrel, n (%)	2603 (53.7)	664 (52.7)	0.371	0.542
ACS (n = 8622)	n = 6005	n = 2617		
Male sex, n (%)	4420 (73.6)	2006 (76.7)	8.916	0.003
Smoking, n (%)	2431 (40.5)	996 (38.1)	4.472	0.034
Drinking, n (%)	1477 (24.6)	530 (20.3)	19.258	<0.01
Age (years)	57.50 ± 11.08	64.10 ± 11.98	26.313	<0.01
BMI (kg/$m^{2}$)	25.88 ± 3.82	25.53 ± 4.11	12.361	0.060
BUN (mmol/L)	8.71 ± 31.00	9.16 ± 18.39	8.398	0.483
Uric acid (mmol/L)	427.33 ± 579.38	440.79 ± 395.74	18.442	0.279
SCr (µmol/L)	88.17 ± 444.01	96.78 ± 68.86	0.547	0.362
eGFR (mL/min/1.73 $m^{2}$)	101.78 ± 31.72	82.93 ± 43.12	333.321	<0.01
TC (mmol/L)	3.93 ± 1.10	3.74 ± 1.07	0.786	<0.01
HDL-C (mmol/L)	1.07 ± 0.31	0.99 ± 0.30	2.254	<0.01
LDL-C (mmol/L)	2.51 ± 0.90	2.39 ± 0.85	6.205	<0.01
LVEF (%)	62.22 ± 5.19	52.70 ± 10.78	2994.446	<0.01
LVEDD (mm)	49.04 ± 4.20	53.78 ± 7.72	1238.945	<0.01
Cys-C (mg/L)	0.84 ± 0.17	1.57 ± 0.95	1201.891	<0.01
ARB or ACEI, n (%)	2450 (40.8)	1243 (47.5)	33.393	<0.01
β-Blockers, n (%)	3275 (56.9)	1412 (57.7)	0.438	0.508
CCB, n (%)	1103 (19.2)	413 (16.9)	5.879	0.015
Aspirin, n (%)	5671 (94.4)	2419 (92.4)	12.642	<0.01
Statins, n (%)	5605 (93.3)	2346 (89.6)	34.660	<0.01
Clopidogrel, n (%)	2966 (49.4)	1335 (51.0)	1.914	0.166

Abbreviations: BMI, body mass index; SCr, serum creatinine; BUN, blood urea nitrogen; eGFR, estimated glomerular filtration rate; TC, total cholesterol; HDL-C, high-density lipoprotein cholesterol; LDL-C, low-density lipoprotein cholesterol; LVEF, left ventricular ejection fraction; LVEDD, left ventricular end diastolic diameter; ARB, angiotensin receptor blocker; ACEI, angiotensin-converting enzyme inhibitor; CCB, calcium channel blocker; SCAD, stable coronary artery disease; Cys-C, Cystatin C; ACS, acute coronary syndrome; CLR, Cys-C/LVEF ratio.

### 3.3 Clinical Outcomes

For the primary endpoints, as shown in Table 2, ACM occurred in 443 patients out of the total population during the follow-up. Among the low-CLR group, ACM occurred in 100 (0.9%) patients, while it occurred in 343 (8.8%) patients among the high-CLR group, and highly incidence of ACM was determined in the high-CLR group compared to the low-CLR group (*p* < 0.001). Furthermore, 329 patients had CM: 69 (0.6%) in low-CLR group, 260 (6.7%) in high-CLR group, and a significant difference was found in the CM incidence between the two groups (*p* < 0.001).

In terms of the secondary endpoints, we also found significant differences for MACEs (5.9% vs. 12.7%, *p* < 0.001), MACCEs (6.7% vs. 13.3%, *p* < 0.001) and NFMIs (0.9% vs. 3.3%, *p* < 0.001) between the two groups.

Subgroup analysis indicated that for SCAD patients, there were significant differences in the incidence of ACM (0.9% vs. 7.5%, *p* < 0.001), CM (0.6% vs. 5.1%, *p* < 0.001), MACEs (5.2% vs. 9.9%, *p* < 0.001) and MACCEs (5.8% vs. 10.9%, *p* < 0.001) between the low-CLR group and high-CLR group. For the ACS patients, we also found significant differences in the incidences of ACM (0.9% vs. 9.5%, *p* < 0.001), CM (0.7% vs. 7.5%, *p* < 0.001), MACEs (6.5% vs. 14.0%, *p* <0.001) and MACCEs (7.5% vs. 14.5%, *p* < 0.001) between these two groups (as shown in Table 2).

**Table 2.** **The primary endpoints of the two groups**.

Outcomes	Low-CLR	High-CLR	χ^2^*/t*	*p*-values
Total (n = 14,733)	n = 10,856	n = 3877		
ACM	100 (0.9)	343 (8.8)	615.344	<0.001
CM	69 (0.6)	260 (6.7)	482.219	<0.001
NFMI	97 (0.9)	129 (3.3)	112.032	<0.001
MACEs	643 (5.9)	492 (12.7)	183.996	<0.001
MACCEs	729 (6.7)	517 (13.3)	161.705	<0.001
SCAD (n = 6111)	n = 4851	n = 1260		
ACM	43 (0.9)	94 (7.5)	197.231	<0.001
CM	29 (0.6)	64 (5.1)	134.040	<0.001
NFMI	208 (4.3)	53 (4.2)	0.016	0.899
MACEs	252 (5.2)	125 (9.9)	38.590	<0.001
MACCEs	280 (5.8)	137 (10.9)	40.933	<0.001
ACS (n = 8622)	n = 6005	n = 2617		
ACM	57 (0.9)	249 (9.5)	390.656	<0.001
CM	40 (0.7)	196 (7.5)	318.755	<0.001
NFMI	296 (4.9)	122 (4.7)	0.283	0.595
MACEs	391 (6.5)	367 (14.0)	128.285	<0.001
MACCEs	449 (7.5)	380 (14.5)	104.045	<0.001

Abbreviations: ACM, all-cause mortality; CM, cardiac mortality; MACEs, major adverse cardiovascular events; MACCEs, major adverse cardiovascular and cerebrovascular events, NFMI, nonfatal myocardial infarction; SCAD, stable coronary artery disease; ACS, acute coronary syndrome; CLR, cystatin C/left ventricular ejection fraction ratio.

### 3.4 Kaplan-Meier Survival Curve Analysis

**Fig. 3.**
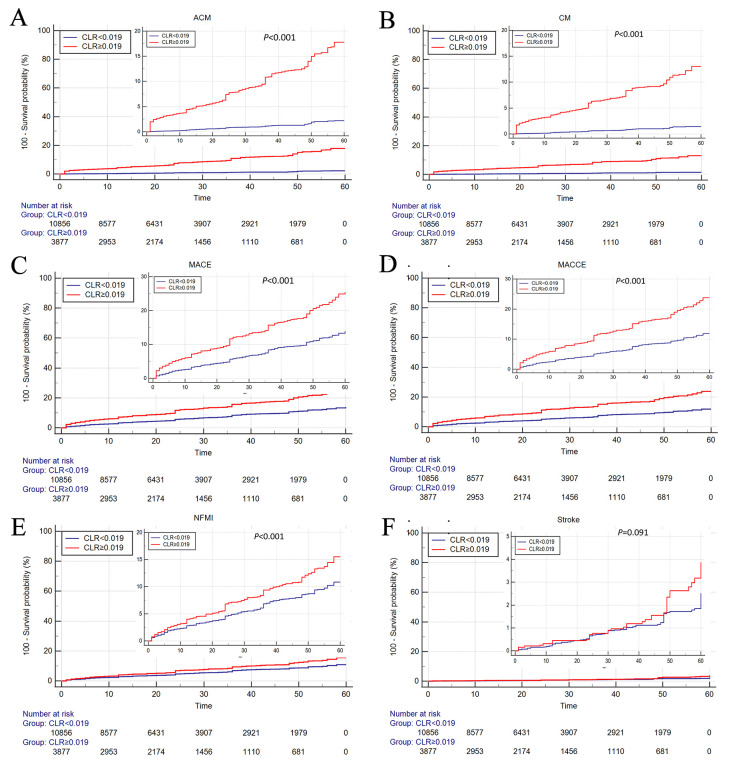
**Cumulative Kaplan‒Meier estimates of the time to the first occurrence of ACM (A), CM (B), MACE (C), MACCE (D), NFMI (E), and stroke (F)**. ACM, all-cause mortality; CM, cardiac mortality; MACE, major adverse cardiovascular event; MACCE, major adverse cardiovascular and cerebrovascular event, NFMI, nonfatal myocardial infarction; CLR, cystatin C/left ventricular ejection fraction ratio.

As shown in Fig. 3, Kaplan-Meier survival analysis was performed to further investigate the effect of CLR on patients’ prognosis. Patients in the high-CLR group (CLR ≥0.019) had higher ACM (A), CM (B), MACEs (C), MACCEs (D), NFMI (E) and stroke (F) rates compare to patients with low-CLR (CLR <0.019) (all *p* < 0.001).

### 3.5 Multivariate Cox Regression Analysis of the Two Groups

We performed multivariate Cox regression analysis after adjusting for age, sex, smoking, drinking, hypertension, diabetes, TC, HDL-C, LDL-C, BMI, therapy with ARB or ACEI, β-Blockers, CCB, aspirin, statins and clopidogrel. Compared to those of the low-CLR group, the risks of ACM, CM, MACEs, MACCEs, and NFMI of the high-CLR group increased 8.163-fold (HR = 8.163, 95% CI: 4.730~14.087, *p* < 0.001), 10.643-fold (HR = 10.643, 95% CI: 5.525~20.501, *p* < 0.001), 2.352-fold (HR = 2.352, 95% CI: 1.754~3.154, *p* < 0.001), 2.137-fold (HR = 2.137, 95% CI: 1.611~2.834, *p* < 0.001), and 1.580-fold (HR = 1.580, 95% CI: 1.273~1.960, *p* < 0.001), respectively (Tables 3,4,5,6,7).

**Table 3.** **Cox regression analysis results for ACM**.

Variables	Beta	SE	Wald	*p*-values	Hazard ratio (95% CI)
Age	0.059	0.012	24.053	<0.001	1.061 (1.036~1.087)
Male sex	–0.664	0.317	4.391	0.036	0.515 (0.277~0.958)
Smoking	–0.361	0.288	1.573	0.210	0.697 (0.396~1.226)
Drinking	0.110	0.325	0.116	0.734	1.117 (0.591~2.110)
Hypertension	0.001	0.289	0.000	0.997	1.001 (0.568~1.763)
Diabetes	0.404	0.252	2.564	0.109	1.497 (0.914~2.454)
TC	0.133	0.231	0.333	0.564	1.143 (0.727~1.797)
HDL-C	–0.188	0.449	0.175	0.675	0.828 (0.343~1.998)
LDL-C	–0.060	0.294	0.041	0.839	0.942 (0.529~1.678)
BMI	–0.055	0.031	3.211	0.073	0.946 (0.891~1.005)
CLR	2.100	0.278	56.879	<0.001	8.163 (4.730~14.087)

TC, total cholesterol; HDL-C, high-density lipoprotein cholesterol; LDL-C, low-density lipoprotein cholesterol; BMI, body mass index; CLR, cystatin C/left ventricular ejection fraction ratio; ACM, all-cause mortality.

**Table 4.** **Cox regression analysis results for CM**.

Variables	Beta	SE	Wald	*p*-values	Hazard ratio (95% CI)
Age	0.048	0.014	12.387	<0.001	1.050 (1.022~1.078)
Male sex	–0.754	0.382	3.896	0.048	0.470 (0.222~0.995)
Smoking	–0.434	0.330	1.725	0.189	0.648 (0.339~1.238)
Drinking	0.321	0.359	0.801	0.371	1.379 (0.682~2.785)
Hypertension	–0.002	0.332	0.000	0.994	0.998 (0.520~1.914)
Diabetes	0.534	0.298	3.210	0.073	1.707 (0.951~3.062)
TC	–0.066	0.384	0.030	0.863	0.936 (0.441~1.985)
HDL-C	0.047	0.528	0.008	0.929	1.048 (0.373~2.948)
LDL-C	0.085	0.460	0.034	0.854	1.088 (0.442~2.680)
BMI	–0.034	0.035	0.957	0.328	0.967 (0.903~1.035)
CLR	2.365	0.334	49.991	<0.001	10.643 (5.525~20.501)

TC, total cholesterol; HDL-C, high-density lipoprotein cholesterol; LDL-C, low-density lipoprotein cholesterol; BMI, body mass index; CLR, cystatin C/left ventricular ejection fraction ratio; CM, cardiac mortality.

**Table 5.** **Cox regression analysis results for NFMI**.

Age	Beta	SE	Wald	*p*-values	Hazard ratio (95% CI)
Male sex	–0.003	0.004	0.460	0.498	0.997 (0.988~1.006)
Smoking	–0.036	0.122	0.088	0.767	0.965 (0.760~1.224)
Drinking	–0.329	0.116	8.049	0.005	0.720 (0.573~0.903)
Hypertension	0.335	0.118	8.104	0.004	1.397 (1.110~1.759)
Diabetes	0.091	0.107	0.724	0.395	1.095 (0.888~1.351)
TC	0.061	0.093	0.433	0.511	1.063 (0.886~1.277)
HDL-C	–0.107	0.113	0.897	0.344	0.899 (0.721~1.121)
LDL-C	0.012	0.172	0.005	0.943	1.012 (0.723~1.418)
BMI	0.130	0.131	0.979	0.322	1.139 (0.880~1.472)
CLR	0.457	0.110	17.223	<0.001	1.580 (1.273~1.960)

TC, total cholesterol; HDL-C, high-density lipoprotein cholesterol; LDL-C, low-density lipoprotein cholesterol; BMI, body mass index; CLR, cystatin C/left ventricular ejection fraction ratio; NFMI, nonfatal myocardial infarction.

**Table 6.** **Cox regression analysis results for MACEs**.

Variables	Beta	SE	Wald	*p*-values	Hazard ratio (95% CI)
Age	0.004	0.007	0.392	0.531	1.004 (0.991~1.017)
Male sex	–0.462	0.194	5.668	0.017	0.630 (0.431~0.922)
Smoking	–0.271	0.165	2.695	0.101	0.762 (0.551~1.054)
Drinking	0.215	0.171	1.587	0.208	1.240 (0.887~1.732)
Hypertension	0.160	0.165	0.941	0.332	1.173 (0.850~1.621)
Diabetes	0.404	0.142	8.118	0.004	1.497 (1.134~1.976)
TC	0.112	0.095	1.390	0.238	1.119 (0.928~1.349)
HDL-C	–0.346	0.265	1.710	0.191	0.707 (0.421~1.189)
LDL-C	–0.035	0.127	0.074	0.785	0.966 (0.753~1.239)
BMI	0.001	0.017	0.001	0.970	1.001 (0.967~1.035)
CLR	0.855	0.150	32.621	<0.001	2.352 (1.754~3.154)

TC, total cholesterol; HDL-C, high-density lipoprotein cholesterol; LDL-C, low-density lipoprotein cholesterol; BMI, body mass index; CLR, cystatin C/left ventricular ejection fraction ratio; MACEs, major adverse cardiovascular events.

**Table 7.** **Cox regression analysis results for MACCEs**.

Variables	Beta	SE	Wald	*p*-values	Hazard ratio (95% CI)
Age	0.006	0.006	1.065	0.302	1.007 (0.994~1.019)
Male sex	–0.430	0.180	5.698	0.017	0.650 (0.457~0.926)
Smoking	–0.304	0.158	3.692	0.055	0.738 (0.541~1.006)
Drinking	0.265	0.163	2.659	0.103	1.304 (0.948~1.793)
Hypertension	0.255	0.160	2.545	0.111	1.291 (0.943~1.766)
Diabetes	0.271	0.133	4.163	0.041	1.311 (1.011~1.700)
TC	0.095	0.099	0.918	0.338	1.100 (0.905~1.335)
HDL-C	–0.185	0.245	0.568	0.451	0.831 (0.514~1.344)
LDL-C	–0.015	0.128	0.013	0.910	0.986 (0.766~1.268)
BMI	–0.004	0.017	0.048	0.827	0.996 (0.964~1.029)
CLR	0.759	0.144	27.776	<0.001	2.137 (1.611~2.834)

TC, total cholesterol; HDL-C, high-density lipoprotein cholesterol; LDL-C, low-density lipoprotein cholesterol; BMI, body mass index; CLR, cystatin C/left ventricular ejection fraction ratio; MACCEs, major adverse cardiovascular and cerebrovascular events.

## 4. Discussion

We clarified, in the present study, that CAD patient with high-CLR who received PCI have a worse survival over 5 years compare to those with low-CLR. Besides, elevated CLR was confirmed to be an independent predictor of ACM, CM, MACEs and MACCEs for both SCAD and ACS patients underwent PCI. And this is the first study to reveal the association between CLR and adverse outcomes in CAD patients, as far as we know.

As a latent cysteine protease inhibitor, Cys-C plays a crucial part in human vascular pathophysiology [22]. High levels of Cys-C in serum were previously considered to be independently connected to the incidence of cardiovascular events, even among patients considered to be low risk for renal function dysfunction [23, 24, 25]. Cys-C, therefore, was regarded as a potential biomarker for cardiovascular disease. Furthermore, there is increasing evidence suggesting that Cys-C is a effective predictor of prognosis, whether CAD patients are undergoing coronary revascularization or not [26, 27, 28]. According to the study of Wallentin *et al*. [29], Cys-C has been linked to cardiovascular events and death among CAD patients. In additon, evaluated levels of Cys-C in serum were linked to increased long-term ACM and CM risks among STEMI patients in a retrospective study by Chen *et al*. [30]. Taglieri *et al*. [31] demonstrated that enhanced Cys-C levels were relevant to higher mortality risk and incidence of myocardial infarction in patients with ACS. Interestingly, Liu *et al*. [32] found that Cys-C levels in serum were not an independent predictor of long-term mortality among patients following coronary angiography. We determined that Cys-C levels and CAD prognosis were positively correlated in our study, which involved 14,733 patients with CAD patients. Besides, we also assessed the predictive efficacy of the LVEF for CAD outcomes and demonstrated its good discriminability for mortality (AUC = 0.719 for ACM; AUC = 0.739 for CM). Although either Cys-C or LVEF alone is a powerful predictor for mortality, the ratio of Cys-C to LVEF (the CLR) showed better performance in predicting mortality (AUC = 0.819 for ACM; AUC = 0.826 for CM). Therefore, we believe that the CLR is a stronger predictor of adverse outcomes than Cys-C or LVEF alone in CAD patients. Similarly, Serkan Ordu *et al*. [33] found that Cys-C is an independent risk factor for evaluating the prognosis of patients with chronic heart failure. When LVEF <35%, Cys-C has a stronger predictive value for the prognosis of adverse events. It seems to indirectly verify the correlation between Cys-C and LVEF to some extent.

Furthermore, compare to patients with low CLR, those with high CLR are prominently more likely to experience ACM, CM, NFMI, MACEs and MACCEs. The two groups differed significantly in many baseline characteristics, including age, sex, smoking, drinking, hypertension, diabetes, TC, HDL-C, LDL-C, and BMI. Taking these confounders into account, multivariable Cox regressions was performed, which shows that the incidence of ACM, CM, MACEs, and MACCEs remarkably strengthened in patients with high-CLR compared to those with low-CLR, and this result was more pronounced in SCAD and ACS patients after PCI. Therefore, those results are credible and likely not incidental. The association between an increased CLR and adverse outcomes may be explained by several potential pathophysiological mechanisms. A previous study [34] indicated that patients with higher Cys-C levels have a higher metabolic state, and Cys-C is a fruitful inhibitor of lysosomal protease and cysteine protease was produced by almost all human cells at a constant rate [35]. A high Cys-C concentration may promote inflammation, regulate oxidative stress, and release more cytokines [36]. In addition, a reduced LVEF suggests the presence of heart failure, which indicates that the patient has a poor prognosis. Therefore, a combined analysis of these two parameters may improve the predictive ability for a CAD prognosis.

There are several strengths in our study. First, the AUC and HR values of the CLR were considerably higher than those independent of Cys-C or LVEF indicators, making the CLR highly innovative. Second, the CLR was observed to be associated with outcomes in CAD for the first time in current study, which increases the strength of our claims. Third, a prospective cohort with a large number of patients was constructed in this study, which improved its statistical power. Fourth, we performed multivariable regression analyses, thus improving the reliability and generalizability of our results. Nonetheless, there are still several limitations in our research. First, we only collected baseline serum Cys-C and LVEF data, and dynamic changes in these two parameters were not available in current study. Second, since this study is a single-centre study, a multicentre study was needed to confirm those results.

## 5. Conclusions

In summary, in the current study we indicated that an elevated serum Cys-C to LVEF ratio was significantly associated with a poor prognosis in patients with CAD who underwent PCI and that it shows effective predictive value in SCAD and ACS patients. Hence, CLR might be a novel and credible indicator of mortality and adverse events among CAD patients. Furthermore, it might be helpful to distinguish patients at high risk of cardiovascular disease through CLR.

## Data Availability

The datasets used and/or analyzed during the current study available from the corresponding author on reasonable request.
